# Helpers compensate for age‐related declines in parental care and offspring survival in a cooperatively breeding bird

**DOI:** 10.1002/evl3.213

**Published:** 2021-01-20

**Authors:** Martijn Hammers, Sjouke A. Kingma, Lotte A. van Boheemen, Alexandra M. Sparks, Terry Burke, Hannah L. Dugdale, David S. Richardson, Jan Komdeur

**Affiliations:** ^1^ Groningen Institute for Evolutionary Life Sciences University of Groningen Groningen CP 9712 The Netherlands; ^2^ Department of Animal Science Wageningen University & Research Wageningen PB 6708 The Netherlands; ^3^ School of Biological Sciences Monash University Clayton VIC 3800 Australia; ^4^ School of Biology University of Leeds Leeds LS2 9JT United Kingdom; ^5^ Department of Animal and Plant Sciences University of Sheffield Sheffield S10 2TN United Kingdom; ^6^ School of Biological Sciences University of East Anglia Norwich NR4 7TJ United Kingdom; ^7^ Nature Seychelles Mahé Republic of Seychelles

**Keywords:** Ageing, cooperative breeding, parental care, senescence, sociality

## Abstract

Offspring from elderly parents often have lower survival due to parental senescence. In cooperatively breeding species, where offspring care is shared between breeders and helpers, the alloparental care provided by helpers is predicted to mitigate the impact of parental senescence on offspring provisioning and, subsequently, offspring survival. We test this prediction using data from a long‐term study on cooperatively breeding Seychelles warblers (*Acrocephalus sechellensis*). We find that the nestling provisioning rate of female breeders declines with their age. Further, the total brood provisioning rate and the first‐year survival probability of offspring decline progressively with age of the female breeder, but these declines are mitigated when helpers are present. This effect does not arise because individual helpers provide more care in response to the lower provisioning of older dominant females, but because older female breeders have recruited more helpers, thereby receiving more overall care for their brood. We do not find such effects for male breeders. These results indicate that alloparental care can alleviate the fitness costs of senescence for breeders, which suggests an interplay between age and cooperative breeding.

Senescence—the progressive age‐dependent decline in reproductive performance and survival, for example, as a result of a decline in physiological condition—occurs in a wide variety of organisms (Nussey et al. 2013; Jones et al. 2014). An increasing number of studies have investigated how age‐dependent declines in reproduction and survival are shaped by environmental conditions, individual characteristics, and trade‐offs between early‐life reproductive investment and late‐life performance (Nussey et al. 2013; Lemaître et al. 2015; Cooper and Kruuk 2018). However, surprisingly little is known about how, and to what extent, the fitness costs of senescence are influenced by an individual's social environment (Bourke 2007; Hammers et al. 2015; Blumstein et al. 2018).

In many cooperatively breeding species, care for offspring is shared between the dominant breeding pair and a variable number of sexually mature subordinate helpers that provide alloparental care (Cockburn 1998). The amount of care that dominant breeders provide is often reduced when they are assisted by helpers and this lower parental investment may improve their own survival and future reproductive output (Hatchwell 1999; Heinsohn 2004). Furthermore, the alloparental care provided by helpers may reduce offspring mortality when the total amount of care delivered to the offspring is increased (Hatchwell 1999; Heinsohn 2004), as has been shown in many species, including humans (Sear and Mace 2008).

Negative associations between parental age and the survival of offspring are frequently observed in wild animals (e.g., Descamps et al. 2008; Hayward et al. 2009). Such age‐dependent declines in offspring survival could arise because elderly parents produce offspring that are of lower intrinsic (e.g., genetic) quality (Kern et al. 2001) or are less able to provide sufficient parental care. Although several studies have provided evidence for late‐life changes in foraging behavior and declines in foraging success (MacNulty et al. 2009; Lecomte et al. 2010; Zimmer et al. 2011), there is a surprising lack of studies that have investigated how the amount of parental care provided to the offspring changes in older individuals. Furthermore, if the ability to provide parental care declines with age, the care provided to offspring by helpers might alleviate the negative effects of parental senescence on parental care and offspring survival in cooperatively breeding species. Such an effect may occur because helpers provide more care in response to a reduction in the parental workload of the dominants, or because older dominants recruit more helpers. Although the few studies that have tested this prediction in mammals found no support for it (Sharp and Clutton‐Brock 2010; Stahler et al. 2013), studies on other taxonomic groups with different modes of reproduction (e.g., birds and insects) are lacking. Clearly, further studies are needed to determine whether cooperation can buffer the adverse effects of senescence.

In this study, we investigated the impact of parental age and alloparental care on the rate of food provisioning to offspring and on first‐year survival of offspring. This was done using the long‐term individual‐level dataset collected on the Cousin Island population of the Seychelles warbler (*Acrocephalus sechellensis*). In this facultatively cooperative‐breeding bird species, the pair‐bonded dominant breeding pair (dominants) often have one or two subordinates of either sex in their territory. Some of these subordinates help the dominants with various aspects of reproductive duties, including provisioning offspring (Komdeur 1994). This is an excellent system to study the effects of cooperative breeding and senescence on offspring survival because the almost complete absence of emigration means that mortality is not confounded by dispersal, and because extrinsic mortality is low due to a lack of predation on adults. This, combined with intensive monitoring resulting in high annual resighting rates, means that individuals can be followed throughout their entire lives (Hammers et al. 2015). Furthermore, because only one third of all subordinates help (Hammers et al. 2019), it is possible to disentangle the benefits of help from group size (Hammers et al. 2019; van Boheemen et al. 2019), which is challenging, or impossible, in many cooperatively breeding species (e.g., subordinates may be the result rather than the cause of high reproductive success [Cockburn et al. 2008]).

Here, we test whether nestling provisioning rate and offspring survival decline with the age of male and female dominant breeders, and test whether helpers mitigate such declines. Our results suggest that alloparental care alleviates the fitness costs of senescence for female breeders and their offspring, which suggests an interplay between age and cooperative breeding.

## Methods

### STUDY DESIGN

The Seychelles warbler population on Cousin Island (29 ha; 4°20′ S, 55°40′ E) has been monitored since 1985. We used data collected between 1994 and 2016, when the population was most intensively studied. Each year, the population contains approximately 320 color‐ringed adult individuals (>96% of individuals have been ringed since 1997) of known sex and age in approximately 115 territories (Richardson et al. 2001). The warbler's life history is characterized by high annual adult survival (84%), mostly single‐egg clutches, and a long period of offspring dependency for a small passerine (up to 3 months) (Komdeur 1994; Brouwer et al. 2006). Individuals that have acquired a dominant breeding position generally defend the same territory, with the same partner, until their death. However, the correlation between the age of the dominant male and female in a territory is weak (this study: *r* (184) = 0.17, *P =* 0.022), because individual dominants that die are replaced by a younger individual (Hammers et al. 2019). Male and female dominants have similar breeding tenure duration, annual survival probabilities, and rates of survival senescence (Brouwer et al. 2006; Hammers et al. 2013). Although Seychelles warblers can breed year‐round, the majority of breeding activity occurs in June‐September (hereafter: main breeding season), when food availability is highest (breeding occurs in 94% of territories in this period (Komdeur 1991). Female subordinates often (44% of female subordinates) lay an egg in the same nest as the dominant female (Richardson et al. 2001). Extra‐group paternity is common; approximately 40% of offspring are sired by a dominant male from outside the breeding group, whereas subordinate males very rarely obtain paternity and extra‐group maternity (i.e., conspecific brood parasitism) does not occur (Richardson et al. 2001).

All territories were checked for the presence of color‐ringed individuals each year during the main breeding season. Any unringed individuals were caught using mist nets and given a combination of three color rings and a British Trust for Ornithology metal ring. The age of individuals was determined based on the long‐term demographic data and eye color (Komdeur 1991). As the annual resighting probability is high (0.97 for dominants and 0.83 for juveniles and subordinates [Brouwer et al. 2012]), and the emigration rate is very low (0.10%; 29), we could confidently assume that individuals that were not observed for two consecutive years had died in the first year that they were not seen. The dominance status of individuals (dominant or subordinate) in each territory was determined from behavioral interactions (affiliative behavior and mate‐guarding) during regular territory visits during the breeding season. We checked each territory for breeding activity at least once every 2 weeks by following the resident dominant female for at least 15 min. Once a nest was found, breeding attempts were monitored every 3‐4 days until the nestling(s) fledged or the breeding attempt failed. To establish whether a subordinate provided nest care (helper) or not (nonhelping subordinate), we conducted nest watches of at least 60 min (max. 90 min) during incubation and on approximately day 10 (mean ± SE = 9.81 ± 0.31, *n* = 186) of the nestling provisioning stage (Bebbington et al. 2017; Hammers et al. 2019). These nest watches were performed throughout the day (0700h to 1800h) and we avoided doing observations in rainy or very windy conditions that could potentially affect provisioning behavior. We used the nest watches performed during the nestling provisioning stage (*n* = 186 nests; 156 nests were observed once and 15 were observed twice) to assess provisioning rates of all individuals that provided care (i.e., the dominants and any helping subordinates). Previous work on Seychelles warblers has shown that provisioning rates observed at the same nest across the nestling period are repeatable (*r* ^2^ = 0.45), suggesting that our observation regime is sufficient to produce a representative measure of provisioning rate at a given nest (Bebbington et al. 2017). We recorded the number of provisioning events (i.e., each food delivery to the nestling) by each provisioning individual in the territory (i.e., the dominant female, dominant male, and any subordinates of either sex). Because the aim of these nest watches was to establish whether any subordinates that were present in a territory helped, and to quantify the amount of help provided by these subordinates, these nest watches are more often conducted in territories with subordinates than in territories without subordinates. This does not pose a problem for the purpose of our study, as the inclusion of a relatively higher number of territories with subordinates does not systematically bias our assessment of help on provisioning per se. The dataset on offspring first‐year survival does not have this selection as nests without subordinates (and thus no potential helpers) could be included, even when no nest watch was performed.

### DATA SELECTION

For our provisioning rate analyses, we used nest watches from the main breeding season where the individuals bringing food to the nest were identified in >90% of provisioning events. We excluded watches where nestlings were still being brooded, as brooding and food provisioning are mutually exclusive behaviors. Brooding occurs mainly during the first week after hatching, although brooding can also occur later in the provisioning period to protect the nestling against unfavorable weather conditions. For our analyses of offspring first‐year survival, we used data from nestlings and fledglings that hatched during the main breeding season and for which the identity of the genetic parents could be assigned with at least 80% confidence (mean ± SE confidence of paternity in our dataset was 0.99 ± 0.001, *n* = 297) based on 30 microsatellites using Masterbayes 2.52 (Hadfield et al. 2006). These offspring were first caught and ringed as a nestling (10‐17 days’ old) or as a fledgling (18‐day to 3‐month old) within their natal territory. Offspring that were ringed outside the main breeding period were excluded because the lower fieldwork intensity in periods outside the main breeding season means that the age and first‐year survival cannot be estimated as reliably for these individuals. To avoid sibling competition confounding our results (Bebbington et al. 2017), we only included nests that contained a single nestling (87% of nests have a clutch size of a single egg [Komdeur 1991]) and, for first‐year survival, only nestlings and fledglings that originated from nests with a single nestling. Subordinate females may be the sole female parent at their nest (Richardson et al. 2002), and because we were interested in the survival of offspring in relation to the age of the dominants, we excluded nestlings and fledglings that resulted from eggs laid by subordinate females (*n* = 31).

### STATISTICAL ANALYSES

We used generalized linear mixed models (GLMM) with either a Poisson error structure and log link function (individual and total provisioning rate), or with a binomial error structure and logit link function (offspring first‐year survival). The GLMMs were fitted with the package *lme4* version 1.1‐12 (Bates et al. 2015) in R version 3.2.5 (R Core Team 2017). We checked for collinearity between the fixed effects by calculating Variance Inflation Factors (VIF). As all VIF were <3, collinearity was not an issue in our analyses. As variables are often on very different scales, and to aid interpretation of the model coefficients (e.g., in the presence of interaction terms), continuous predictor variables were standardized prior to analyses to a mean of zero and a standard deviation of 0.5 (Gelman 2008). All main effects remained in the models, irrespective of the significance of their effect. Nonsignificant (i.e., *P >* 0.05) interactions between main effects were removed from the models, starting with interactions involving quadratic terms. This was done sequentially, in order of least significance.

### PROVISIONING RATES

First, we determined the impact of the dominant's age and the presence of helping subordinates on the provisioning rates of dominant females and males. In the majority of territories with helpers there is only a single helper of either sex (one helper: 86%; two helpers: 14%; three helpers: <1% [Hammers et al. 2019]), therefore we treated helper presence as a binary variable (Y/N). As the number or the sex of helpers may also affect provisioning rates or offspring first‐year survival, we repeated the final models by replacing the binary factor helper presence with (a) the number of helpers or (b) the presence/absence of male (Y/N) and/or female (Y/N) helpers. We then investigated whether these models explained the data better (by comparing the AICc values of these models) than a model with helper presence per se. We included as random effects in the models the identity of the dominant male or female to control for repeated observations of the same dominants, and year, to control for unmeasured annual variation. To account for potential overdispersion, we also included an observation‐level random effect. Because provisioning nest watches varied in duration, we included the log of nest watch duration as an offset in the analyses. Age of the male or female dominant, the quadratic effects of age (age^2^), and helper presence (Y/N) were included as predictors. We also included the interactions between age (and age^2^) of the dominant and helper presence to test the prediction that the slope of the relationship between the dominant's age and offspring provisioning rates changes depending on helper presence. The number of subordinates in the territory (i.e., both helping and nonhelping subordinates) was included as a predictor to disentangle the impact of help from subordinate presence per se (Hammers et al. 2019; van Boheemen et al. 2019). Chick age (days from hatching) and time of day (hour) were included as predictors as these variables may affect provisioning rates. Then, for a subset of dominants for which the age of death was known (i.e., individuals that died within the study period and individuals that were not translocated to other islands as part of an ongoing conservation program), we also included the age of death of the focal dominant as a predictor in the model. This accounts for the potential selective disappearance of lower quality individuals with a shorter lifespan (van de Pol and Verhulst 2006). The results of models that also included the age of death as a predictor did not differ qualitatively from those that did not (Table S1), so the results are reported without age of death to maximize statistical power. Finally, in a separate analysis we added the provisioning rates of the partner and the helpers (instead of the binary variable helper presence) as explanatory variables to the analyses of the dominant female and male provisioning rate to investigate whether an age‐dependent change in provisioning may be explained by the provisioning of the partner or the helpers.

Second, we investigated how the total provisioning rate to a nestling (the total brood provisioning rate), which is the sum of the provisioning rates of all group members, varied with age of the dominants and helper presence. The predictors in this analysis were the same as in the analysis of the dominant's provisioning rate, except that the ages of both the male and the female dominant were included as predictors and that both male and female dominant identity were included as random effects. In addition, the interactions between age (and age^2^) and helper presence were included for both the male and the female dominant.

Third, for the subset of territories that had one or more helpers (*n* = 81, 64 × 1 helper, 16 × 2 helpers, and 1 × 3 helpers), we investigated how the provisioning rate of the helpers in the territory was related to age and the provisioning rates of the dominants, the number of helpers, and the number of subordinates. We did this for all helpers in the territory combined and for the provisioning rate per helper. Finally, we investigated whether the likelihood of having more than one helper was related to the age of the dominant male or female. For this, we performed a binomial GLM with the ages of the dominant male and female as predictors.

### OFFSPRING FIRST‐YEAR SURVIVAL

First‐year survival was a binary variable stating whether a nestling/fledgling survived until one year after the season in which it hatched. We investigated whether survival of offspring is related to the age of the dominant female, the dominant male, and helper presence. Year was included as a random effect. We did not include dominant male and female identity as random effects because the variance that was explained by these variables was zero. Dominant male and female age and age^2^, helper presence, the number of subordinates, the sex of the nestling/fledgling, and the interactions between dominant (fe)male age (and (fe)male age^2^) and helper presence were included as predictors. In addition, we included a binary variable stating whether an individual was first caught as a nestling or as a fledgling, as age at the first catch is positively associated with first‐year survival (because fledglings have already survived the nestling stage; see Table 3). Similar as for the analysis of provisioning rates, the result of this model did not differ qualitatively when we refitted this model on a subset of the data for which the age of death was known for both dominants to account for selective disappearance effects (Table S2).

### ETHICS STATEMENT

The work was conducted with the permission of the Seychelles Bureau of Standards and the Seychelles Ministry of Environment, Energy and Climate Change and complied with all local ethical guidelines and regulations.

## Results

### PROVISIONING RATES

**Figure 1:**
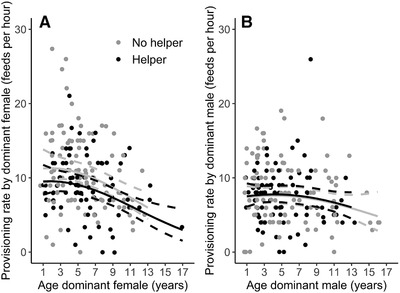
Provisioning rates to offspring in relation to helper presence for (A) dominant female and (B) dominant male Seychelles warblers. Data points are raw data. Lines are model‐predicted regression slopes ± 95% CI from the models in Table 1.

Provisioning rates of dominant females to nestlings were on average 24% higher than those of dominant males (mean ± SE = 9.78 ± 0.34 vs. 7.88 ± 0.30 feeds per hour) during the same observation session (Wilcoxon matched‐pairs test: *V* = 10,882, *P* < 0.001, *n* = 186; Fig. 1). Overall, provisioning rates of dominant males and females were weakly, but positively correlated (*r* (184) = 0.19, *P* = 0.008).

The provisioning rates of dominant females, with and without helpers, declined progressively with their age (Table 1 and Fig. 1). Overall, dominant females with a helper had 16% lower provisioning rates (mean ± SE = 8.85 ± 0.44 [*n* = 81] feeds per hour with help vs. 10.49 ± 0.49 [*n* = 105] without help; Table 1 and Fig. 1). Provisioning rates were not associated with the total number of subordinates (i.e., helpers and nonhelpers) that were present in the territory (Table 1). The effects of dominant female age and helper presence remained significant when we accounted for selective disappearance effects (Table S1) or the provisioning rate of the partner (Table S3), and we found a negative relationship between dominant female provisioning rate and provisioning rate of the helpers (Table S3). Nonetheless, the interaction between dominant female age (and age^2^) and helper presence was not significant (Table 1). Female helpers in territories with a single helper showed higher provisioning rates than male helpers (6.23 ± 0.49 feeds per hour [*n* = 44] vs. 4.47 ± 0.62 [*n* = 20]; Wilcoxon rank sum test: *w* = 587.5, *P* = 0.033). However, dominant female provisioning rate was not better explained by models that included the presence of male and female helpers (ΔAICc = 4.06), or the number of helpers (ΔAICc = 1.26), instead of helper presence as a binary variable (Table S4).

**Table 1:** Provisioning rates of dominant female (A) and male (B) Seychelles warblers in relation to age of the dominants and helper presence. Statistically significant variables are in bold and underlined

(A) Dominant female					(B) Dominant male			
	Estimate	SE	*z*	*P*	Estimate	SE	*z*	*P*
Intercept	**2.35**	**0.06**	**36.85**	**<0.001**	**2.03**	**0.08**	**26.44**	**<0.001**
Age dominant	−**0.25**	**0.08**	−**2.95**	**0.003**	−0.05	0.10	−0.50	0.614
Age^2^ dominant	−0.19	0.12	−1.64	0.102	−0.16	0.16	−1.04	0.299
Helper (Y/N)	−**0.17**	**0.08**	−**2.07**	**0.038**	0.02	0.09	0.22	0.824
Number of subordinates	0.03	0.09	0.30	0.762	−**0.22**	**0.10**	−**2.30**	**0.022**
Chick age	0.00	0.07	−0.01	0.992	−0.10	0.07	−1.36	0.173
Time of day	0.00	0.07	−0.01	0.991	0.12	0.07	1.71	0.088
Age dominant × helper	0.16	0.15	1.02	0.309	−0.02	0.17	−0.10	0.920
Age^2^ dominant × helper	0.08	0.24	0.32	0.751	−0.09	0.35	−0.27	0.791
Random	Variance	*N*			Variance	*N*		
Observation ID	0.02	186			0.05	186		
Dominant ID	0.08	132			0.06	131		
Year	0.01	18			<0.01	18		

In contrast to dominant females, provisioning rates of dominant males were not significantly associated with their age and helper presence (Table 1 and Fig. 1), although provisioning rates were positively associated with helper provisioning rate (Table S3). The provisioning rates of dominant males were positively associated to the provisioning rates of the partner and there was some evidence that the magnitude of this effect is related to male age (Table S3). Provisioning rates were lower when more subordinates were present in the territory (Table 1).

**Figure 2:**
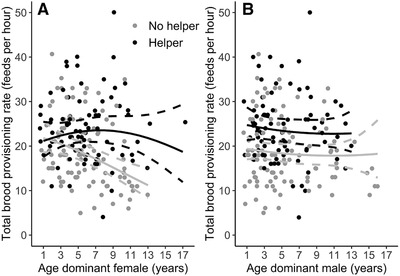
Total provisioning rates to offspring by all feeders in territories with (black) and without helpers (gray) present in relation to the age of (A) dominant female and (B) dominant male Seychelles warblers. Data points are raw data. Lines are model predicted regression slopes ± 95% CI from the model in Table 2.

The total brood provisioning rate (i.e., by all feeders at the nest combined) was, on average, 26% higher in territories with helpers than in territories without (mean ± SE = 23.95 ± 7.65 [*n* = 81] vs. 18.96 ± 7.59 [*n* = 105] feeds per hour; Table 2 and Fig. 2). The total provisioning rate declined with age of the dominant female in territories without helpers (Fig. 2; GLMM: β dominant female age ± SE = −0.28 ± 0.07, *z = −*3.86, *n* = 105, *P* < 0.001), but did not significantly decline when helpers were present (Fig. 2; β dominant female age ± SE = −0.01 ± 0.08, *z =* −0.12, *n* = 81, *P* = 0.907). The significant interaction between the age of the dominant female and helper presence (Table 2) indicated that for nests with helpers, the decline in total provisioning rate due to female age was less severe compared to the decline for nests without helpers (Fig. 2). Repeating this analysis with the number of helpers or the presence of male and/or female helpers instead of the binary factor helper presence gave similar results (Table S5), although these additional models were better supported by the data (ΔAICc = −7.41 and −4.16, respectively). The total provisioning rate was not significantly associated with dominant male age or its interaction with helper presence (Table 2 and Fig. 2).

**Table 2:** The total provisioning rates to the offspring (combining all provisioning individuals) in relation to helper presence and age of dominant female and male Seychelles warblers. Statistically significant variables are in bold and underlined

	Estimate	SE	*z*	*P*
Intercept	**2.91**	**0.06**	**52.32**	**<0.001**
Age dominant female	−**0.30**	**0.08**	−**3.87**	**<0.001**
Age^2^ dominant female	−0.10	0.08	−1.20	0.232
Age dominant male	−0.07	0.07	−0.95	0.341
Age^2^ dominant male	0.04	0.11	0.41	0.686
Helper (Y/N)	**0.24**	**0.06**	**3.88**	**<0.001**
Number of subordinates	0.00	0.06	−0.02	0.988
Chick age	−0.07	0.05	−1.29	0.197
Time of day	0.09	0.05	1.77	0.077
Age dominant female × helper	**0.36**	**0.11**	**3.29**	**0.001**
Age^2^ dominant female × helper	0.28	0.19	1.51	0.131
Age dominant male × helper	0.04	0.11	0.40	0.690
Age^2^ dominant male × helper	0.17	0.23	0.73	0.469
Random	Variance	*N*		
Observation ID	0.06	186		
Dominant female ID	<0.01	132		
Dominant male ID	<0.01	131		
Year	<0.01	118		

In territories with helpers, the provisioning rate by all helpers combined increased with the dominant male's provisioning rate and the number of helpers that were present, but it was not related to the age of the dominants or the dominant female's provisioning rate (Table S6). The per capita provisioning rate of individual helpers was not related to age of the dominants (Fig. S1) or to the number of helpers (Table S6). The likelihood of having more than one helper increased with age of the dominant female (Fig. S2; β ± SE = 2.04 ± 0.67, *z* = 3.03, *P* = 0.002), but this did not increase significantly with age of the dominant male (Fig. S2; β ± SE = 1.04 ± 0.61, *z* = 1.69, *P* = 0.091).

### OFFSPRING SURVIVAL

**Figure 3:**
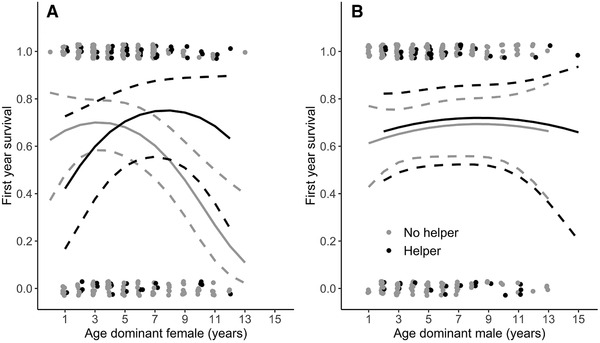
Offspring first‐year survival in territories with (black) and without (gray) helpers in relation to the age of (A) dominant female and (B) dominant male Seychelles warblers. Data points are raw data. Lines are model predicted regression slopes ± 95% CI from the model in Table 3.

When no helpers were present, the first‐year survival probability of offspring declined strongly with age of the dominant female (Table 3 and Fig. 3; GLMM: β dominant female age ± SE = −0.93 ± 0.30, *z = −*3.09, *n* = 242, *P* = 0.002). The quadratic effect of age for dominant females indicated that the rate of this decline becomes steeper for older females (Table 3). Offspring survival was not associated with age of the dominant male (Table 3) and the number of subordinates in the territory. Although, overall, we detected no effect of helpers on offspring survival (Table 3), the significant interaction between dominant female age and helper presence indicated that helpers mitigated, or even reversed, the age‐dependent decline in offspring survival (Table 3 and Fig. 3A). Indeed, in territories with helpers, we found no decline in offspring survival with age of the dominant female (Fig. 3; GLMM: β dominant female age ± SE = 0.61 ± 0.63, *z =* 0.97, *n* = 55, *P* = 0.331). This interaction between age and helper presence remained significant after accounting for the lifespan of the dominants (Table S2). Models that included the number of helpers of the presence of male and female helpers (Table S7) were equally or less well supported by the data (number of helpers: ΔAICc = 0.42; male and female helpers: ΔAICc = 3.64).

**Table 3:** Offspring first‐year survival in relation to helper presence and age of the dominants. Statistically significant variables are in bold and underlined

	Estimate	SE	*z*	*P*
Intercept	−0.49	0.42	−1.18	0.237
Age dominant female	−0.61	0.33	−1.84	0.066
Age^2^ dominant female	−**0.96**	**0.48**	−**1.98**	**0.048**
Age dominant male	0.25	0.34	0.73	0.463
Age^2^ dominant male	−0.24	0.47	−0.50	0.614
Caught as fledgling (vs. nestling)	**1.41**	**0.37**	**3.80**	**<0.001**
Helper (Y/N)	0.13	0.40	0.32	0.752
Offspring sex (male vs. female)	0.39	0.27	1.41	0.159
Number of subordinates	−0.32	0.32	−1.01	0.313
Age dominant female × helper	**1.55**	**0.65**	**2.39**	**0.017**
Age^2^ dominant female × helper	0.04	1.16	0.04	0.969
Age dominant male × helper	−0.88	0.70	−1.25	0.213
Age^2^ dominant male × helper	0.79	1.12	0.70	0.482
Random	Variance	*N*		
Year	0.31	21		
		Total *N* = 297		

## Discussion

We found that nestling provisioning rate of dominant female Seychelles warblers declined with age. This decline was associated with a lower total brood provisioning rate in territories with older dominant females that did not receive help from subordinates. However, the total provisioning rate to nests from older female dominants was not reduced when helpers were present, which indicates that helpers mitigated the age‐dependent decline in provisioning by dominant females. Our results indicate that this compensation by helpers does not arise because helpers actively increase their provisioning rates in the nests of older dominant females, but rather because older dominant females have more helpers. The first‐year survival of offspring from unassisted pairs declined with the age of the dominant female, but the presence of helpers compensated for this decline. We did not find such effects for dominant males.

In the Seychelles warbler, dominant females invest more in parental care than dominant males, as only females incubate the egg and have higher provisioning rates (Komdeur 1994). The lower parental investment of dominant males may potentially be explained by the high levels of extra‐group paternity in this species (Richardson et al. 2001). Males with lower confidence in paternity may be predicted to provide parental care at a rate that is well below their maximum sustainable rate (Dixon et al. 1994; Schroeder et al. 2016, but see Sheldon 2002). The sex that invests more in parental care is predicted to show a higher rate of senescence in parental care (Fay et al. 2016). The steep age‐dependent decline in provisioning in dominant females, compared to no such decline in dominant males, in our study concurs with this prediction. One explanation for this greater decline in dominant females than in males is that females may not be able to maintain their initially higher provisioning rate (Fig. 1) when age‐related declines in physiological condition occur. In addition, the fact that young dominant females show higher provisioning rates than young dominant males means that there is more scope for age‐dependent declines in provisioning rates for dominant females. An age‐dependent decline in provisioning rate might also occur in response to a higher provisioning rate by the other individuals in the territory when the dominant female is older. However, in our study the age‐dependent decline in provisioning was not explained by such a “load‐lightening” effect as the age‐dependent decline still occurred after statistically controlling for the provisioning rates of the dominant male and helpers. Provisioning rate as used in our study may not adequately reflect the total amount of food that is delivered to the nestling if the quality or quantity of the food changes with age of the dominant female. For example, an age‐related decline in provisioning rate might also result from an age‐related increase in foraging efficiency (e.g., bringing more, or more nutritious, food per provisioning event), rather than from an age‐related decline in foraging efficiency. However, our result that offspring survival also declines with female age suggests that this is not the case in Seychelles warblers. Nonetheless, future studies should also consider prey type and quantity to test this possibility. In contrast, incubation attendance—another energetically demanding aspect of parental care—does not decline in older females in the Seychelles warbler (Hammers et al. 2019). An explanation for this may be that there is strong selection against reductions in incubation attendance because lower incubation attendance is associated with a higher risk of egg predation and thus failure of the entire reproductive attempt in this species (Komdeur and Kats 1999).

We expected that offspring survival would be higher in territories with helpers, as was found in earlier studies (Komdeur 1994; Brouwer et al. 2012). Contrary to this expectation, we found that effects of helper presence on offspring survival were only apparent when older dominant females resided in the territory. This difference between the current study and earlier studies might be explained by increases in habitat quality and productivity over time. During the early stages of the overall Seychelles warbler study, higher quality territories were more likely to have helpers than lower quality territories and offspring were also more likely to survive in those higher quality territories (Komdeur 1991; Komdeur 1992). However, these effects were not detected in studies that analyzed more recent data from the Seychelles warbler (Komdeur and Pels 2005; Eikenaar et al. 2010), possibly as a consequence of habitat restoration on the island that has led to a drastic decrease in among‐territory variation in territory quality, an overall increase in territory quality, and an island‐wide increase in productivity.

Previous studies on the Seychelles warbler have shown that higher nestling provisioning rates in this species are associated with higher nestling body mass and with higher nestling and fledgling survival (Komdeur 1991; Komdeur 1994; Bebbington et al. 2018). Our finding—that first‐year survival of offspring from unassisted pairs declined with the age of the dominant female, but that the presence of helpers compensated for this decline—is thus probably the consequence of the increased amounts of parental care provided to the nestlings in broods where helpers were present. However, as offspring can be provisioned for up to 4 months after fledging (Komdeur 1991), postfledgling care by helpers is also likely to contribute to this effect.

The finding that helpers compensate for senescent declines in provisioning rate and offspring survival of dominant females extends the results of a previous study on Seychelles warblers (Hammers et al. 2019), which showed that having helpers was associated with higher late‐life survival and delayed senescence for dominant females. A similar effect has also been described for Alpine marmots (*Marmota marmota*) (Berger et al. 2018). Such late‐life fitness benefits of breeding cooperatively lead to the prediction that older dominant females should be more inclined to recruit helpers to improve their survival and reproduction. In the Seychelles warbler, female subordinates are more likely to become a helper and provide more help than males, and the likelihood that female subordinate females help increases sharply with age of the dominant female (Hammers et al. 2019). Therefore, older dominant females may be predicted to produce more female offspring, although this prediction remains to be tested. In the current study, the likelihood of having more than one helper, but not the provisioning rate per helper, was positively associated with age of the dominant female. This indicates that the compensation benefits provided by helpers do not arise through an active process where helpers provision more in direct response to the lower provisioning of older dominant females, but rather through a more passive process where older females are more likely to have multiple helpers (Fig. S3). Future studies may test if helpers alleviate the fitness costs of parental senescence in other cooperatively breeding species and explore the possibility that dominants strategically recruit helpers to mitigate the impact of senescence, which may lead to more cooperative breeding behavior among elderly individuals. Furthermore, future studies may further investigate the effect of the age (Cooper et al. 2020) and sex of helpers on provisioning and offspring survival.

Our results suggest that the improvement in late‐life fitness associated with cooperative breeding may lead to selection on helping behavior and longer lifespan. Our study also illustrates that to reveal and understand the factors that shape variation in senescence rates, as well as the evolutionary forces behind the maintenance of cooperation, it may be important to apply a fine‐scale assessment of the context in which these processes occur.

## AUTHOR CONTRIBUTIONS

MH designed the study, wrote the manuscript, and analyzed the data. All authors performed research, including specifically fieldwork (MH, DSR, SAK, and LAB) and compilation of the dataset (MH, HLD, and LAB). All authors provided input into concepts and ideas, provided feedback on the analyses, and critically revised and edited the manuscript. DSR, JK, TB, HLD, MH, and SAK acquired funding. DSR, JK, TB, and HLD coordinated the long‐term study.

## CONFLICT OF INTEREST

The authors declare no conflict of interest.

Associate Editor: A. Gardner

## Supporting information


**Table S1**. Provisioning rates of dominant female (A) and male (B) Seychelles warblers in relation to their age and helper presence.
**Table S2**. Offspring first‐year survival in relation to helper presence and age of the dominants.
**Table S3**. Provisioning rates of dominant female (A) and male (B) Seychelles warblers in relation to their age and to the provisioning rate of their partner and helpers.
**Table S4**. Provisioning rates of dominant female Seychelles warblers in relation to their age and (A) the presence of male or female helpers and (B) the number of helpers.
**Table S5**. The total provisioning rates to the offspring (combining all provisioning individuals) in relation to the age of the dominants and (A) the presence of male or female helpers and (B) the number of helpers.
**Table S6**. Provisioning rates of helpers in relation to the age of the dominants and provisioning rate of the dominants.
**Table S7**. Offspring first‐year survival in relation to age of the dominants and (A) the presence of male or female helpers and (B) the number of helpers.
**Figure S1**. Provisioning rates to offspring by helpers in relation to (a) age of the dominant female and (b) age of the dominant male.
**Figure S2**. The likelihood of having more than one helper in relation to (a) age of the dominant female and (b) age of the dominant male.
**Figure S3**. Provisioning rates to offspring by different numbers of helpers in relation to (a) age of the dominant female and (b) age of the dominant male.

## Data Availability

All data are available in the Dryad depository: https://doi.org/10.5061/dryad.rxwdbrv4s (Hammers et al. 2020)
