# Supplementary material for: Helpers compensate for age‐related declines in parental care and offspring survival in a cooperatively breeding bird
Source: Evol Lett. 2021 Jan 20;5(2):143–53. doi: 10.1002/evl3.213 (PMC8045936; doi:10.1002/evl3.213)

**Electronic Supplementary Materials**

M. Hammers, S. A. Kingma, L. A. van Boheemen, A. M. Sparks, T. Burke, H. L. Dugdale, D. S. Richardson and J. Komdeur. Helpers compensate for age-related declines in parental care and offspring survival in a cooperatively breeding bird.

**Supplementary Table 1:** Provisioning rates of dominant female (A) and male (B) Seychelles warblers in relation to their age and helper presence. To account for selective disappearance effects, we included the age of death of the focal dominant. Statistically significant variables are in bold and underlined.

|  |  |  |  |  |  |  |  |  |  |
| --- | --- | --- | --- | --- | --- | --- | --- | --- | --- |
| A) dominant female |  |  |  |  |  | B) dominant male |  |  |  |
|  | Estimate | SE | *z* | *P* |  | Estimate | SE | *z* | *P* |
| Intercept | **2.34** | **0.07** | **32.80** | **<0.001** |  | **2.05** | **0.08** | **24.75** | **<0.001** |
| Age dominant | **-0.41** | **0.13** | **-3.17** | **0.002** |  | -0.23 | 0.15 | -1.53 | 0.127 |
| Age^2^ dominant | -0.22 | 0.13 | -1.62 | 0.104 |  | -0.10 | 0.17 | -0.61 | 0.543 |
| Helper (Y/N) | **-0.23** | **0.10** | **-2.41** | **0.016** |  | -0.07 | 0.11 | -0.66 | 0.506 |
| Age of death dominant | 0.22 | 0.12 | 1.81 | 0.070 |  | **0.28** | **0.13** | **2.16** | **0.031** |
| Number of subordinates | 0.04 | 0.09 | 0.40 | 0.693 |  | -0.19 | 0.11 | -1.79 | 0.073 |
| Chick age | 0.00 | 0.08 | -0.05 | 0.958 |  | -0.08 | 0.08 | -0.96 | 0.339 |
| Time of day | 0.01 | 0.08 | 0.08 | 0.938 |  | 0.11 | 0.08 | 1.37 | 0.170 |
| Age dominant x helper | 0.19 | 0.18 | 1.03 | 0.303 |  | 0.12 | 0.20 | 0.63 | 0.528 |
| Age^2^ dominant x helper | 0.12 | 0.29 | 0.42 | 0.672 |  | 0.11 | 0.40 | 0.26 | 0.794 |
|  |  |  |  |  |  |  |  |  |  |
| Random | Variance | N |  |  |  | Variance | N |  |  |
| Observation ID | 0.01 | 153 |  |  |  | 0.07 | 152 |  |  |
| Dominant ID | 0.10 | 112 |  |  |  | 0.05 | 108 |  |  |
| Year | 0.01 | 18 |  |  |  | <0.01 | 18 |  |  |
|  |  |  |  |  |  |  |  |  |  |

**Supplementary Table 2:** Offspring first-year survival in relation to helper presence and age of the dominants. The age of death dominants is included to account for selective disappearance effects. Statistically significant variables are in bold and underlined.

|  |  |  |  |  |
| --- | --- | --- | --- | --- |
|  | Estimate | SE | *z* | *P* |
| Intercept | -0.37 | 0.45 | -0.81 | 0.418 |
| Age dominant female | -0.60 | 0.43 | -1.40 | 0.161 |
| Age^2^ dominant female | -1.03 | 0.53 | -1.93 | 0.054 |
| Age dominant male | -0.28 | 0.46 | -0.62 | 0.534 |
| Age^2^ dominant male | -0.17 | 0.51 | -0.34 | 0.736 |
| Caught as fledgling (vs. nestling) | **1.18** | **0.39** | **3.01** | **0.003** |
| Helper (Y/N) | 0.35 | 0.44 | 0.81 | 0.417 |
| Offspring sex (male vs. female) | 0.47 | 0.30 | 1.53 | 0.126 |
| Number of subordinates | -0.30 | 0.35 | -0.86 | 0.390 |
| Age of death dominant female | -0.01 | 0.38 | -0.04 | 0.971 |
| Age of death dominant male | 0.67 | 0.41 | 1.66 | 0.097 |
| Age dominant female x helper | **2.13** | **0.74** | **2.87** | **0.004** |
| Age^2^ dominant female x helper | 0.38 | 1.30 | 0.29 | 0.771 |
| Age dominant male x helper | -1.38 | 0.80 | -1.74 | 0.082 |
| Age^2^ dominant male x helper | 1.66 | 1.33 | 1.25 | 0.211 |
|  |  |  |  |  |
| Random | Variance | N |  |  |
| Year | 0.40 | 21 |  |  |
|  |  | Total N: 250 |  |  |
|  |  |  |  |  |

**Supplementary Table 3:** Provisioning rates of dominant female (A) and male (B) Seychelles warblers in relation to their age and to the provisioning rate of their partner and helpers.

|  |  |  |  |  |  |  |  |  |  |
| --- | --- | --- | --- | --- | --- | --- | --- | --- | --- |
| A) dominant female |  |  |  |  |  | B) dominant male |  |  |  |
|  | Estimate | SE | *z* | *P* |  | Estimate | SE | *z* | *P* |
| Intercept | **2.26** | **0.05** | **41.73** | **<0.001** |  | **2.05** | **0.06** | **35.97** | **<0.001** |
| Age dominant | **-0.23** | **0.08** | **-2.78** | **0.006** |  | -0.02 | 0.10 | -0.20 | 0.842 |
| Age^2^ dominant | -0.16 | 0.12 | -1.37 | 0.170 |  | -0.26 | 0.17 | -1.55 | 0.122 |
| Provisioning rate helper | **-0.18** | **0.08** | **-2.25** | **0.024** |  | **0.18** | **0.09** | **2.12** | **0.034** |
| Provisioning rate partner | **0.18** | **0.07** | **2.64** | **0.008** |  | **0.40** | **0.11** | **3.71** | **<0.001** |
| Number of subordinates | 0.06 | 0.08 | 0.74 | 0.459 |  | **-0.29** | **0.09** | **-3.22** | **0.001** |
| Chick age | 0.01 | 0.07 | 0.20 | 0.839 |  | -0.08 | 0.07 | -1.17 | 0.241 |
| Time of day | -0.01 | 0.07 | -0.14 | 0.892 |  | 0.09 | 0.07 | 1.38 | 0.168 |
| Age dominant x provisioning rate helper | 0.08 | 0.14 | 0.59 | 0.556 |  | 0.19 | 0.15 | 1.23 | 0.219 |
| Age^2^ dominant x provisioning rate helper | -0.09 | 0.17 | -0.52 | 0.607 |  | -0.51 | 0.35 | -1.44 | 0.150 |
| Age dominant x provisioning rate partner | 0.01 | 0.16 | 0.07 | 0.942 |  | 0.30 | 0.19 | 1.54 | 0.123 |
| Age^2^ dominant x provisioning rate partner | -0.31 | 0.31 | -1.00 | 0.317 |  | **-0.79** | **0.37** | **-2.15** | **0.031** |
|  |  |  |  |  |  |  |  |  |  |
| Random | Variance | N |  |  |  | Random | Variance | N |  |
| Observation ID | 0.02 | 186 |  |  |  | Observation ID | 0.04 | 186 |  |
| Dominant ID | 0.07 | 132 |  |  |  | Dominant ID | 0.06 | 131 |  |
| Year | 0.01 | 18 |  |  |  | Year | <0.01 | 18 |  |
|  |  |  |  |  |  |  |  |  |  |

**Supplementary Table 4:** Provisioning rates of dominant female Seychelles warblers in relation to their age and (A) the presence of male or female helpers and (B) the number of helpers. Statistically significant variables are in bold and underlined.

|  |  |  |  |  |
| --- | --- | --- | --- | --- |
| A) dominant female provisioning, male and female helpers | | |  |  |
|  | Estimate | SE | *z* | *P* |
| Intercept | **2.33** | **0.06** | **36.56** | **<0.001** |
| Age dominant | **-0.24** | **0.08** | **-2.90** | **0.004** |
| Age^2^ dominant | -0.18 | 0.12 | -1.52 | 0.129 |
| Female helper (Y/N) | -0.13 | 0.09 | -1.44 | 0.149 |
| Male Helper (Y/N) | -0.08 | 0.10 | -0.88 | 0.379 |
| Number of subordinates | 0.02 | 0.09 | 0.23 | 0.820 |
| Chick age | 0.01 | 0.07 | 0.08 | 0.935 |
| Time of day | 0.00 | 0.07 | -0.02 | 0.986 |
|  |  |  |  |  |
| Random | Variance | N |  |  |
| Observation ID | 0.03 | 186 |  |  |
| Dominant ID | 0.08 | 132 |  |  |
| Year | 0.01 | 18 |  |  |
|  |  |  |  |  |
| B) dominant female provisioning, number of helpers | |  |  |  |
|  | Estimate | SE | *z* | *P* |
| Intercept | **2.27** | **0.06** | **40.74** | **<0,001** |
| Age dominant | **-0.23** | **0.08** | **-2.82** | **0.005** |
| Age^2^ dominant | -0.18 | 0.12 | -1.55 | 0.121 |
| Number of helpers | -0.15 | 0.09 | -1.73 | 0.084 |
| Number of subordinates | 0.03 | 0.09 | 0.32 | 0.751 |
| Chick age | 0.01 | 0.07 | 0.11 | 0.916 |
| Time of day | 0.00 | 0.07 | 0.07 | 0.942 |
|  |  |  |  |  |
| Random | Variance | N |  |  |
| Observation ID | 0.02 | 186 |  |  |
| Dominant ID | 0.07 | 132 |  |  |
| Year | 0.01 | 18 |  |  |
|  |  |  |  |  |

**Supplementary Table 5:** The total provisioning rates to the offspring (combining all provisioning individuals) in relation to the age of the dominants and (A) the presence of male or female helpers and (B) the number of helpers. Statistically significant variables are in bold and underlined.

|  |  |  |  |  |
| --- | --- | --- | --- | --- |
| A) total provisioning by all individuals, male and female helpers | | |  |  |
|  | Estimate | SE | *z* | *P* |
| Intercept | **2.89** | **0.06** | **50.46** | **<0.001** |
| Age dominant female | **-0.30** | **0.07** | **-4.07** | **<0.001** |
| Age^2^ dominant female | -0.19 | 0.09 | -2.08 | 0.038 |
| Age dominant male | -0.11 | 0.07 | -1.52 | 0.127 |
| Age^2^ dominant male | 0.10 | 0.11 | 0.88 | 0.381 |
| Female helper (Y/N) | **0.29** | **0.07** | **4.17** | **<0.001** |
| Male Helper (Y/N) | **0.18** | **0.07** | **2.43** | **0.015** |
| Number of subordinates | -0.06 | 0.07 | -0.84 | 0.399 |
| Chick age | -0.06 | 0.05 | -1.13 | 0.260 |
| Time of day | **0.10** | **0.05** | **1.97** | **0.049** |
| Age dominant female x female helper | **0.30** | **0.11** | **2.62** | **0.009** |
| Age dominant female x male helper | **0.29** | **0.14** | **2.16** | **0.031** |
|  |  |  |  |  |
| Random | Variance | N |  |  |
| Observation ID | 0.05 | 186 |  |  |
| Dominant female ID | <0.01 | 132 |  |  |
| Dominant male ID | <0.01 | 131 |  |  |
| Year | 0.01 | 118 |  |  |
|  |  |  |  |  |
| B) total provisioning by all individuals, number of helpers | | |  |  |
|  | Estimate | SE | *z* | *P* |
| Intercept | **3.02** | **0.05** | **66.57** | **<0.001** |
| Age dominant female | **-0.17** | **0.06** | **-2.87** | **0.004** |
| Age^2^ dominant female | -0.17 | 0.09 | -1.89 | 0.059 |
| Age dominant male | -0.10 | 0.07 | -1.45 | 0.147 |
| Age^2^ dominant male | 0.06 | 0.11 | 0.59 | 0.554 |
| Number of helpers | **0.29** | **0.07** | **4.17** | **<0.001** |
| Number of subordinates | -0.06 | 0.07 | -0.89 | 0.375 |
| Chick age | -0.07 | 0.05 | -1.33 | 0.184 |
| Time of day | 0.07 | 0.05 | 1.46 | 0.144 |
| Age dominant female x number of helpers | **0.31** | **0.10** | **2.97** | **0.003** |
|  |  |  |  |  |
| Random | Variance | N |  |  |
| Observation ID | 0.05 | 186 |  |  |
| Dominant female ID | <0.01 | 132 |  |  |
| Dominant male ID | <0.01 | 131 |  |  |
| Year | <0.01 | 118 |  |  |
|  |  |  |  |  |

**Supplementary Table 6:** Provisioning rates of helpers in relation to the age of the dominants and provisioning rate of the dominants. A: total provisioning rate of all helpers combined, B: provisioning rates per helper. Statistically significant variables are in bold and underlined.

|  |  |  |  |  |  |  |  |  |  |
| --- | --- | --- | --- | --- | --- | --- | --- | --- | --- |
| A) Total provisioning rate all helpers combined | |  |  |  |  | B) Provisioning rate per helper | |  |  |
|  | Estimate | SE | *z* | *P* |  | Estimate | SE | *z* | *P* |
| Intercept | **1.76** | **0.09** | **19.83** | **<0.001** |  | **1.61** | **0.09** | **17.37** | **<0.001** |
| Age dominant female | -0.09 | 0.15 | -0.58 | 0.565 |  | -0.10 | 0.15 | -0.68 | 0.498 |
| Age^2^ dominant female | 0.01 | 0.15 | 0.07 | 0.944 |  | 0.02 | 0.16 | 0.10 | 0.918 |
| Age dominant male | -0.11 | 0.14 | -0.77 | 0.444 |  | -0.12 | 0.15 | -0.80 | 0.422 |
| Age^2^ dominant male | 0.38 | 0.23 | 1.68 | 0.092 |  | 0.37 | 0.24 | 1.54 | 0.124 |
| Provisioning rate dominant female | -0.13 | 0.12 | -1.14 | 0.256 |  | -0.16 | 0.12 | -1.29 | 0.199 |
| Provisioning rate dominant male | **0.34** | **0.09** | **3.69** | **<0.001** |  | **0.32** | **0.11** | **2.97** | **0.003** |
| Number of helpers | **0.69** | **0.12** | **5.59** | **<0.001** |  | 0.10 | 0.14 | 0.69 | 0.489 |
| Number of subordinates | 0.03 | 0.14 | 0.19 | 0.846 |  | 0.00 | 0.14 | 0.02 | 0.983 |
| Chick age | -0.16 | 0.10 | -1.53 | 0.127 |  | -0.15 | 0.11 | -1.38 | 0.167 |
| Time of day | 0.19 | 0.11 | 1.75 | 0.080 |  | 0.13 | 0.12 | 1.08 | 0.281 |
|  |  |  |  |  |  |  |  |  |  |
| Random | Variance | N |  |  |  | Variance | N |  |  |
| Observation ID | <0.01 | 81 |  |  |  | <0.01 | 81 |  |  |
| Dominant female ID | <0.01 | 59 |  |  |  | <0.01 | 59 |  |  |
| Dominant male ID | 0.10 | 60 |  |  |  | 0.10 | 60 |  |  |
| Year | <0.01 | 17 |  |  |  | <0.01 | 17 |  |  |
|  |  | Total N: 81 |  |  |  |  | Total N: 81 |  |  |
|  |  |  |  |  |  |  |  |  |  |

**Supplementary Table 7:** Offspring first-year survival in relation to age of the dominants and (A) the presence of male or female helpers and (B) the number of helpers. Statistically significant variables are in bold and underlined.

|  |  |  |  |  |
| --- | --- | --- | --- | --- |
| A) male and female helpers |  |  |  |  |
|  | Estimate | SE | *z* | *P* |
| Intercept | -0.49 | 0.42 | -1.19 | 0.235 |
| Age dominant female | -0.57 | 0.33 | -1.69 | 0.091 |
| Age^2^ dominant female | -0.83 | 0.48 | -1.71 | 0.088 |
| Age dominant male | 0.26 | 0.34 | 0.76 | 0.448 |
| Age^2^ dominant male | -0.37 | 0.48 | -0.76 | 0.447 |
| Caught as fledgling (vs. nestling) | **1.49** | **0.38** | **3.95** | **<0.001** |
| Offspring sex (male vs. female) | 0.34 | 0.28 | 1.23 | 0.220 |
| Number of subordinates | -0.26 | 0.32 | -0.79 | 0.428 |
| Male helper (Y/N) | 0.04 | 0.51 | 0.08 | 0.940 |
| Female helper (Y/N) | 0.17 | 0.50 | 0.34 | 0.731 |
| Age dominant female x male helper | **2.48** | **1.06** | **2.34** | **0.019** |
| Age dominant male x female helper | 0.42 | 0.81 | 0.52 | 0.602 |
|  |  |  |  |  |
| Random | Variance | N |  |  |
| Year | 0.32 | 21 |  |  |
|  |  | Total N: 297 |  |  |
|  |  |  |  |  |
| B) number of helpers |  |  |  |  |
|  | Estimate | SE | *z* | *P* |
| Intercept | -0.46 | 0.41 | -1.14 | 0.255 |
| Age dominant female | -0.33 | 0.31 | -1.06 | 0.288 |
| Age^2^ dominant female | **-0.98** | **0.48** | **-2.03** | **0.042** |
| Age dominant male | 0.25 | 0.34 | 0.74 | 0.458 |
| Age^2^ dominant male | -0.20 | 0.46 | -0.44 | 0.663 |
| Caught as fledgling (vs. nestling) | **1.39** | **0.37** | **3.75** | **<0.001** |
| Offspring sex (male vs. female) | 0.39 | 0.27 | 1.42 | 0.155 |
| Number of subordinates | -0.35 | 0.32 | -1.09 | 0.275 |
| Number of helpers | 0.10 | 0.33 | 0.29 | 0.771 |
| Age dominant female x number of helpers | **1.09** | **0.51** | **2.13** | **0.034** |
|  |  |  |  |  |
| Random | Variance | N |  |  |
| Year | 0.31 | 21 |  |  |
|  |  | Total N: 297 |  |  |
|  |  |  |  |  |

**Supplementary Figure 1: Provisioning rates to offspring by helpers in relation to (a) age of the dominant female and (b) age of the dominant male.** Data points are raw data. Lines are model predicted regression slopes ± 95% CI.


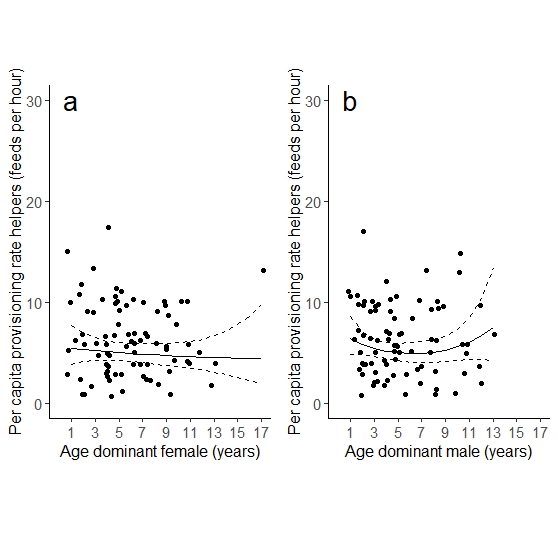


**Supplementary Figure 2: The likelihood of having more than one helper in relation to (a) age of the dominant female and (b) age of the dominant male.** Data points are raw data. Lines are model predicted regression slopes ± 95% CI.


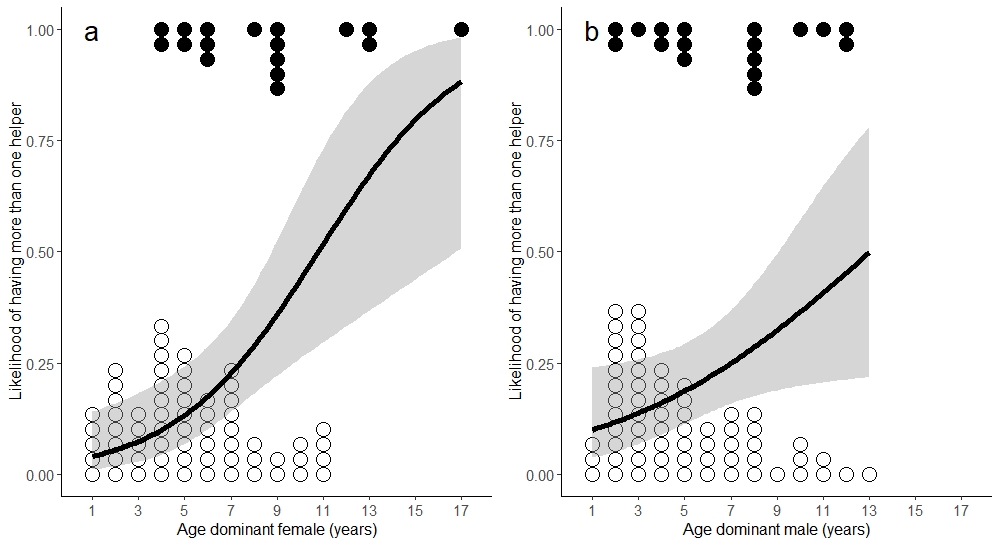


**Supplementary Figure 3:** Provisioning rates to offspring by different numbers of helpers in relation to (a) age of the dominant female and (b) age of the dominant male. Data points are raw data. Bi-directional error bars are 95% CI. Different symbols correspond to different numbers of helpers.


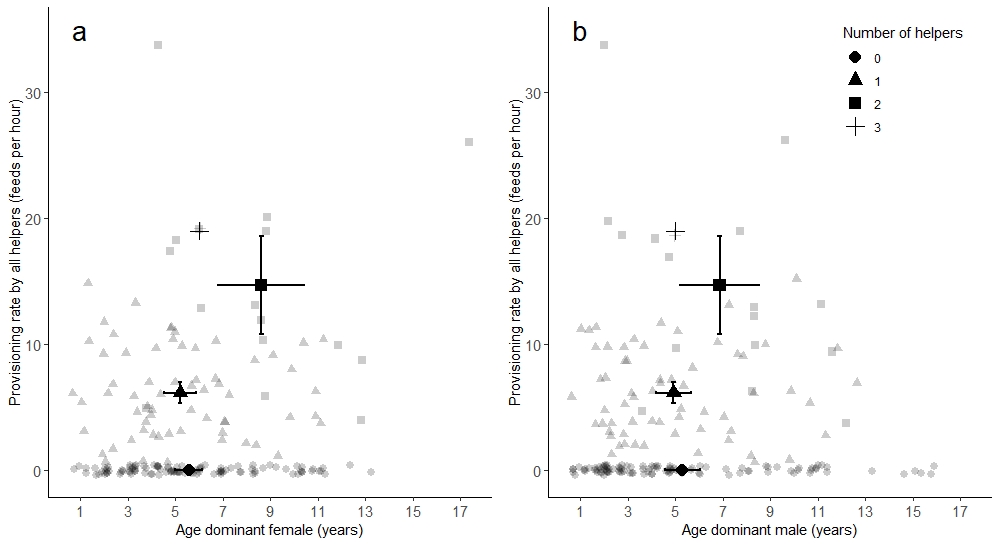

Supplement: Supplementary file 1 — Table S1. Provisioning rates of dominant female (A) and male (B) Seychelles warblers in relation to their age and helper presence. Table S2. Offspring first‐year survival in relation to helper presence and age of the dominants. Table S3. Provisioning rates of dominant female (A) and male (B) Seychelles warblers in relation to their age and to the provisioning rate of their partner and helpers. Table S4. Provisioning rates of dominant female Seychelles warblers in relation to their age and (A) the presence of male or female helpers and (B) the number of helpers. Table S5. The total provisioning rates to the offspring (combining all provisioning individuals) in relation to the age of the dominants and (A) the presence of male or female helpers and (B) the number of helpers. Table S6. Provisioning rates of helpers in relation to the age of the dominants and provisioning rate of the dominants. Table S7. Offspring first‐year survival in relation to age of the dominants and (A) the presence of male or female helpers and (B) the number of helpers. Figure S1. Provisioning rates to offspring by helpers in relation to (a) age of the dominant female and (b) age of the dominant male. Figure S2. The likelihood of having more than one helper in relation to (a) age of the dominant female and (b) age of the dominant male. Figure S3. Provisioning rates to offspring by different numbers of helpers in relation to (a) age of the dominant female and (b) age of the dominant male. [file EVL3-5-143-s001.docx]
